# Rationally Designed Peptidomimetic Modulators of Aβ Toxicity in Alzheimer's Disease

**DOI:** 10.1038/srep08139

**Published:** 2015-01-30

**Authors:** K. Rajasekhar, S. N. Suresh, Ravi Manjithaya, T. Govindaraju

**Affiliations:** 1 Bioorganic Chemistry Laboratory, New Chemistry Unit, Jawaharlal Nehru Centre for Advanced Scientific Research, Jakkur P.O., Bengaluru 560064, Karnataka, India; 2 Molecular Biology and Genetics Unit, Jawaharlal Nehru Centre for Advanced Scientific Research, Jakkur P.O., Bengaluru 560064, Karnataka, India

## Abstract

Alzheimer's disease is one of the devastating illnesses mankind is facing in the 21^st^ century. The main pathogenic event in Alzheimer's disease is believed to be the aggregation of the β-amyloid (Aβ) peptides into toxic aggregates. Molecules that interfere with this process may act as therapeutic agents for the treatment of the disease. Use of recognition unit based peptidomimetics as inhibitors are a promising approach, as they exhibit greater protease stability compared to natural peptides. Here, we present peptidomimetic inhibitors of Aβ aggregation designed based on the KLVFF (P1) sequence that is known to bind Aβ aggregates. We improved inhibition efficiency of P1 by introducing multiple hydrogen bond donor-acceptor moieties (thymine/barbiturate) at the N-terminal (P2 and P3), and blood serum stability by modifying the backbone by incorporating sarcosine (N-methylglycine) units at alternate positions (P4 and P5). The peptidomimetics showed moderate to good activity in both inhibition and dissolution of Aβ aggregates as depicted by thioflavin assay, circular dichroism (CD) measurements and microscopy (TEM). The activity of P4 and P5 were studied in a yeast cell model showing Aβ toxicity. P4 and P5 could rescue yeast cells from Aβ toxicity and Aβ aggregates were cleared by the process of autophagy.

Alzheimer's disease (AD) is a major contributor of dementia with no clinically accepted treatment to cure or halt its progression^1^. Over the past two decades, tremendous efforts have been devoted to understanding the pathogenesis of AD^2^. Although the detailed mechanism of neurodegeneration encountered in AD is not entirely understood yet, several reports indicate that the fibrillar aggregation of β−amyloid (Aβ) 36−42 peptides and, in particular, highly toxic Aβ42 play a key role in the pathogenesis of AD^3^,^4^,^5^,^6^. The Aβ36−42 peptides are derived from a transmembrane protein called amyloid precursor protein (APP). Amyloidogenic pathway for processing of APP by enzymes β- and γ−secretases lead to the release of Aβ36−42 peptides and their deposition in the brain as plaques^7^. Hence, the development of molecular agents that are capable of inhibiting the Aβ fibril formation or dissolution of the preformed toxic Aβ fibrillar aggregates are key concepts for AD treatment^8^,^9^.

Elucidation of the structural properties of Aβ fibrils in the recent years has enabled the design of inhibitors for fibril formation^10^,^11^,^12^,^13^,^14^,^15^,^16^. The hydrophobic core residues from 11 to 25 in Aβ40/42 is very crucial for their assembly into fibrils, and these short peptide sequences have a recognition ability towards Aβ polypeptides. The pentapeptide sequences KLVFF or LVFFA can recognize Aβ polypeptides and, therefore be used as recognition units in the design of inhibitors for Aβ fibrillization. For example, Tjernberg *et al*.^17^ demonstrated that Aβ_16-20_ binds residues 25 to 35 of Aβ and prevents fibril formation. Soto and co-workers^18^ rationally designed a proline-containing residue (LPFFD), which is known to be a β-sheet breaker and was found to inhibit the fibrillization of Aβ aggregation. KLVFF-conjugated oligolysine or oligoglutamic acid units were useful means of generating binders to Aβ, which resulted in the formation of large aggregates that might lead to reduced cell toxicity^19^,^20^,^21^. A tetramer of KLVFF was designed and was found to inhibit the transformation of Aβ42 soluble oligomers into fibrils and also promoted the dissolution of preformed Aβ42 aggregates^22^. Conjugation of hydrophobic moieties with Aβ recognition unit was also attempted to construct inhibitors of Aβ aggregation. Cholic acid as a hydrophobic moiety at the N-terminal of LVFFA and its D-analog sequence strongly inhibited the Aβ fibrillization^23^. Ferrocene attached at the N-terminal of KLVFF showed inhibitory action towards Aβ42 aggregates^24^. Methylation of amide groups in short recognition peptides is also an effective means of designing Aβ inhibitors. These N-methylated peptides were able to cap growing β-sheets, blocking one face of the Aβ polypeptide from participating in hydrogen bond driven fibrillar aggregation due to lack of amide proton and sterically hindered N-methyl groups. Furthermore, the chemical modifications at the N-terminal and amide N-methylated designed peptides also provided extra stability towards proteases^25^,^26^,^27^. Several N-methylated peptides based on recognition sequence (KLVFF) have been systematically synthesized and analysed for their ability to function as fibrillar inhibitors and their effect on the Aβ toxicity. Digit *et al.*^28^ synthesized an analog peptide D-[chGly-Tyr-chGly-chGly-mLeu] -NH_2_ (ch = cyclohexyl, male = *N*-methyl lysine) to demonstrate its striking inhibitory activity. Introducing N-methyl analogs of natural amino acids at alternating positions of recognition peptide have also shown promising activity in both inhibition and dissolution of Aβ aggregates^29^. A completely synthetic analog of the recognition peptide with N-substituted amino acids (peptides) have been shown to have prominent inhibition activity towards Aβ aggregates^30^. Designing hybrid peptide-peptoid based modulators targeting hydrogen bonding involved in β-sheet formation and subsequent elongation leading to fibrillar aggregates has not been addressed adequately in the literature. Therefore, developing hybrid peptide-peptoid based modulators aiming to target multiple phases of Aβ42 aggregation would provide highly efficient inhibitors.

Another potential approach is through enhancing the phenomenon of aggrephagy. Aggrephagy, a cellular mechanism of selective autophagy, involves degradation of misfolded proteins or aggregates essential for cell homeostasis^31^. Presence of Aβ aggregates down-regulates autophagy, which is known to play a pivotal role in the clearance and neutralizing the toxic effects caused by Aβ. Designed small molecules or peptides which influence autophagy may also act as probable therapeutics^32^. Yeast has been popularly used as a simple model organism in literature to study Aβ toxicity and screen Aβ inhibitors^33^. *Saccharomyces cerevisiae* is a eukaryote and, hence, shares phenomenal homology with the human genome^34^. It also recapitulates the fundamental processes of a human-like transcription, translation and also its metabolism^35^. Yeast model also provides a platform to study the autophagy-based regulation^36^.

In this report, we present effective inhibition of Aβ42 aggregation using hybrid peptide-peptiod modulators based on the core sequences of Aβ peptide (KLVFF). The hybrid peptide-peptoids modulators were designed to act on multiple phases of Aβ42 aggregation by introducing a non-amino acid moiety with multiple hydrogen bond donor-acceptor sites, at the N-terminal to target Aβ42 β-sheet formation. The introduction of peptoid monomers (sarcosine) at alternative positions of the recognition motif (KLVFF) prevents the oligomerization of Aβ42 monomers upon its binding through the face of amino acids. Furthermore, the hybrid peptide-peptoid modulators were anticipated to confer proteolysis resistance to the derived peptidomimetics, thus increasing their biostability and bioavailability (the parent peptide KLVFF contains natural amino acids and is not resistant to endoproteases). Thioflavin T (ThT) binding, assayed by fluorescence spectroscopy, was used to probe Aβ42 fibril formation and effect of peptidomimetic inhibitors on their growth. Circular dichroism (CD) was used to study the effect of inhibitors on the secondary structure of Aβ42 aggregates. The morphological analysis of Aβ42 in the absence and presence of peptidomimetic inhibitors was investigated using transmission electron microscopy (TEM). The structural integrity and stability of inhibitory peptides and peptidomimetics was analyzed in the presence of proteases. Further, inhibitory activity was studied in the yeast (*Saccharomyces cerevisiae*) model, which expresses Aβ42, to assess the ability of peptidomimetics as therapeutic agents and to understand their mechanism of action in reducing Aβ42 toxicity. Thus, we report on the study of structural fine tuning and inhibitory activities of peptidomimetics towards preventing the formation of Aβ42 aggregates and dissolving the preformed toxic aggregates (Fig. 1).

## Results and Discussion

### Design strategy for Peptidomimetics

**Figure 1:**
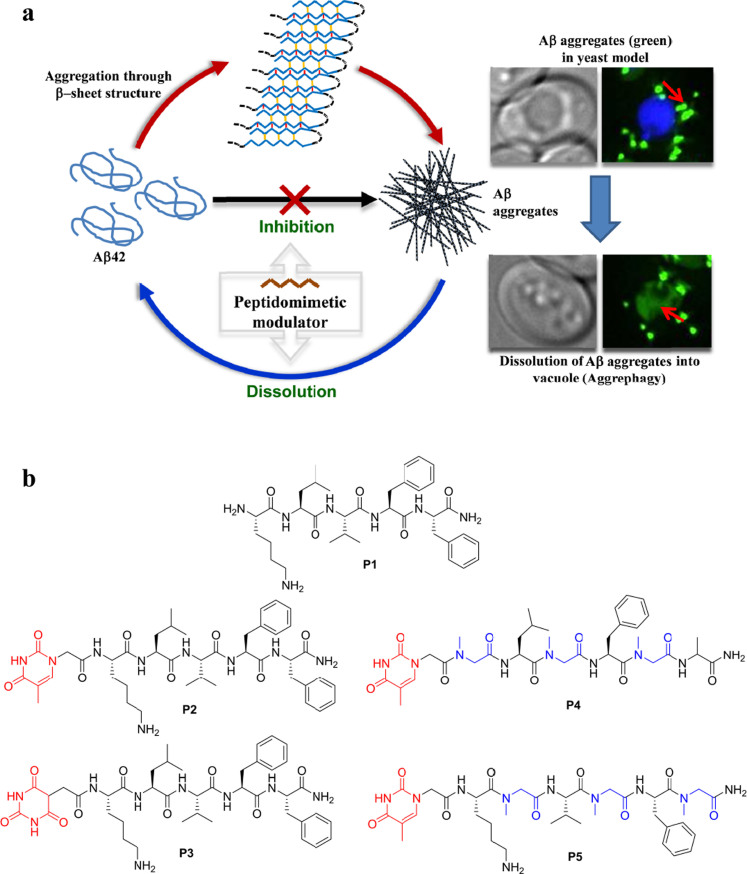
Peptidomimetic inhibitors. (a), Inhibiton and dissolution of Aβ42 aggregates, and their evaluation in yeast model for Alzheimers disease. (b), Structures of peptide and peptidomimetic inhibitors.

The principle of the design was to rationally introduce a minimum number of simple chemical modifications into a small recognition peptide sequence extracted from Aβ42, which is considered crucial for β-sheet conformation and fibrillogenesis. These peptidomimetics bind and stabilize the amyloidogenic conformational population of Aβ42 and inhibit its aggregation into toxic amyloid aggregates. The chemical modifications are aimed at interfering with hydrogen bonding found in the β-sheet conformations of Aβ42^37^. Inhibition of β-sheet formation in Aβ42 affects its self-assembly to fibrillar aggregates. We considered KLVFF (Aβ16-20) as the recognition sequence, which has been reported in the literature to interact with Aβ42 and its aggregates^16^. Although KLVFF (**P1**) has the ability to interfere with fibrillization, the extent of inhibition is very marginal due to higher stabilization of Aβ42 β-sheet conformations than the Aβ42/KLVFF complex^38^. To enhance the stabilization of Aβ42/KLVFF complex we introduced small organic moieties with multiple hydrogen bond donors and acceptors at the N-terminal of KLVFF (Fig. 1). This specially chosen organic moiety could participate in hydrogen bonding to form much stronger Aβ42/inhibitor complex. We selected two organic moieties, thymine and barbiturate, as N-terminal pendent functionalities to obtain peptides **P2** and **P3**, respectively as shown in Fig. 1. These organic moieties contain multiple hydrogen bond donor and acceptor centers, which are capable of forming additional hydrogen bonds with β−sheet forming Aβ42 monomer or Aβ42 aggregates^39^. Subsequently, we performed inhibition studies and concluded that the extent of inhibition was moderate, and moreover, the blood serum or protease stability of **P2** and **P3** was not encouraging. The next level of modification was then considered on **P2** as it displayed better inhibition activity over **P3**. Meredith *et al*. used N-substituted amino acids at alternate positions of KLVFFAE, where α-substituents of Leu, Phe and Ala were attached to amide nitrogen atom. These modifications were presumed to help retain the recognition ability and inhibition of Aβ40 fibrillogenesis or dissolution of Aβ40 fibrils. In this case, the inhibitor was anticipated to work by blocking the hydrogen bonding intereactions. However, involvement of other noncovalent interactions from the α-substituents either in the inhibitor or Aβ40 were not considered in the design^29^. It should be noted that the fibrillogenesis of Aβ40/42 is guided by both hydrogen bonding and other noncovalent interactions^29^. Thus, we intend to target the key role of hydrogen bonding in Aβ42 aggregation as well as minimizing other noncovalent interactions among Aβ42 and modulators, in our design strategy. Keeping this in mind, we introduced sarcosine (Sr) in alternate positions of **P2** to obtain **P4** (Thymine-Sr-Leu-Sr-Phe-Sr-Ala) and **P5** [Thymine-Lys-Sr-Val-Sr-Phe-Sr) (Fig. 1). We hypothesized that the peptidomimetics **P4** and **P5** would interact with Aβ42 through the face containing normal amino acids (blocking hydrogen bonding) while minimizing other noncoavelent interactions to prevent the fibrillogenesis of Aβ42^40^.

### Studying inhibition and dissolution efficiency by thioflavin assay and CD measurements

**Figure 2:**
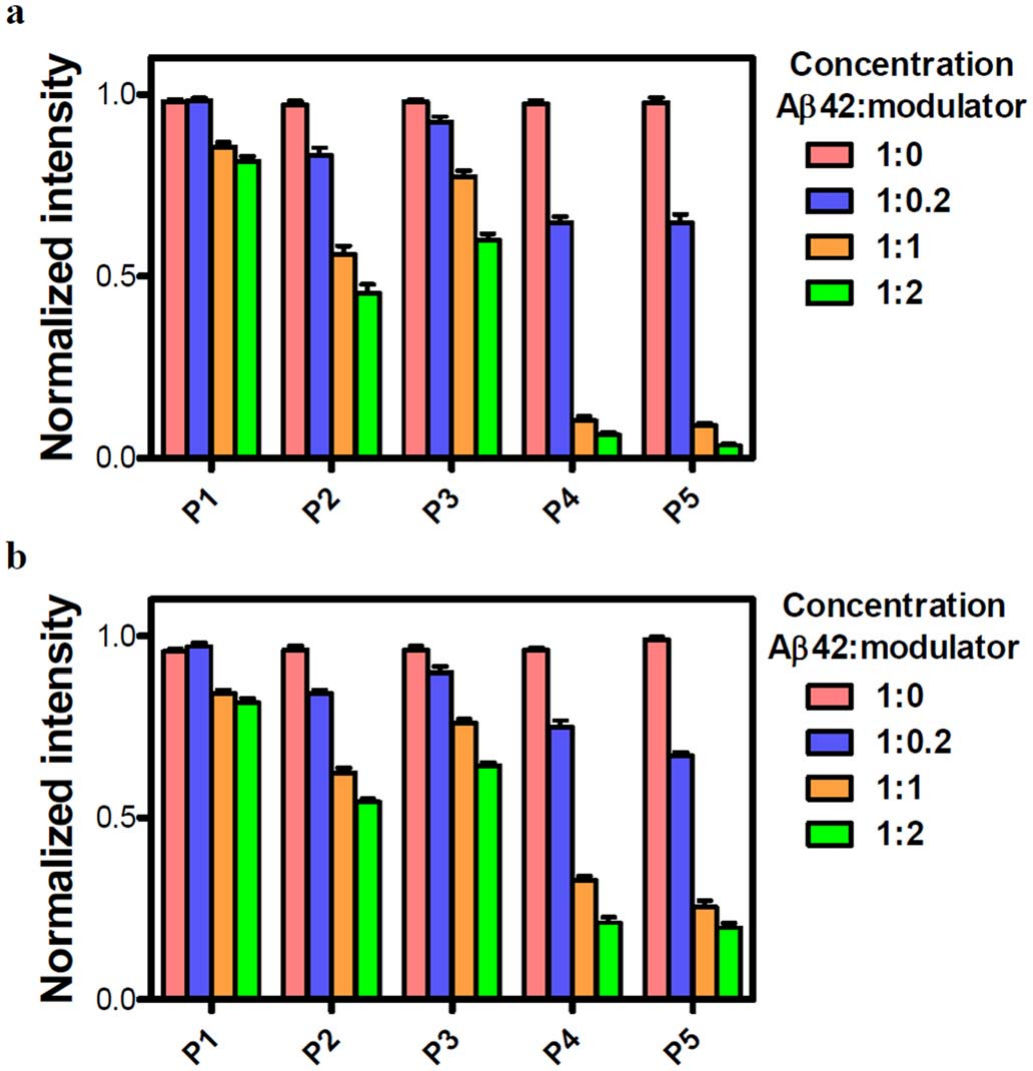
Inhibition and reversal data of Aβ42 aggregates studied by ThT assay. The data in (a) and (b) show the effects of different stoichiometries of **P1**, **P2**, **P3**, **P4** and **P5** on the aggregation of 20 μM Aβ42 (on day 4 for the inhibition assay and day 6 for the reversal assay). Molar ratios (Aβ42:peptide) of 1:0, 1:0.2, 1:1 and 1:2 were used for each peptide. Values are the normalized maximal fluorescence intensity at 485 nm compared to that of the control (Aβ42 with no inhibitor). **P4** and **P5** showed most prominent effect in both the experiments compared to other three peptides (**P1**-**P3**). Each experiment was repeated three times (n = 3). Error bars represent the standard deviation (SD) of the fluorescence measurement.

ThT assay has been widely used to monitor the transformation of Aβ42 monomers to fibrillar aggregates. We employed ThT assay to evaluate the ability of our peptidomimetic candidates to either prevent fibril assembly (inhibition) or to break down preformed fibrils of Aβ42 (dissolution). For the inhibition assay all the peptidomimetics (**P2**, **P3**, **P4** and **P5**) along with control peptide **P1** were added at 0 h of the experiment, whereas for the aggregates reversal (dissolution) assay they were added to Aβ42 fibrillar aggregates grown for 2 days. Once they had been incubated together, Aβ42/inhibitors were analyzed using ThT by measuring the fluorescence changes. First, we performed concentration-dependent experiments where different ratios of **P1**, **P2**, **P3**, **P4** and **P5** were incubated with fixed concentrations of Aβ42 (20 μM) and its aggregates to study their effect on both inhibition and reversal assay, respectively. Experiments were performed at stoichiometric ratios (Aβ42/inhibitor) of 1:0.2, 1:1, and 1:2 with the fixed concentration of Aβ42 of 20 μM. Inhibition experiments demonstrated that **P4** and **P5** were able to prevent Aβ42 aggregation as indicated by a reduction in the fluorescence intensity of ThT up to 95% in case of 1:2 stoichiometric ratio after four days of incubation at 37°C (Fig. 2). Conversely, **P1**, **P2** and **P3** showed low to moderate inhibition efficiencies of 20%, 55% and 40%, respectively for 1:2 stoichiometry. Similar trends were observed in the case of fibril reversal assay with dissolution efficiencies of 20% (**P1**), 45% (**P2**), 34% (**P3**), 80% (**P4**) and 80% (**P5**), for 1:2 stoichiometric ratios (Fig. 2). Thus, **P4** and **P5** were found to be promising as they displayed a pronounced effect on both inhibition and reversal assay. However, the efficiencies of **P4** and **P5** were only marginally better in inhibition assay compared to reversal assay with a difference of about 15%. Increasing the molar ratio of peptides (> 2 fold) did not lead to improvements in inhibition or reversal assay and, therefore, we performed all our further experiments with 1:2 stoichiometry of Aβ42:inhibitor (20 μM:40 μM).

**Figure 3:**
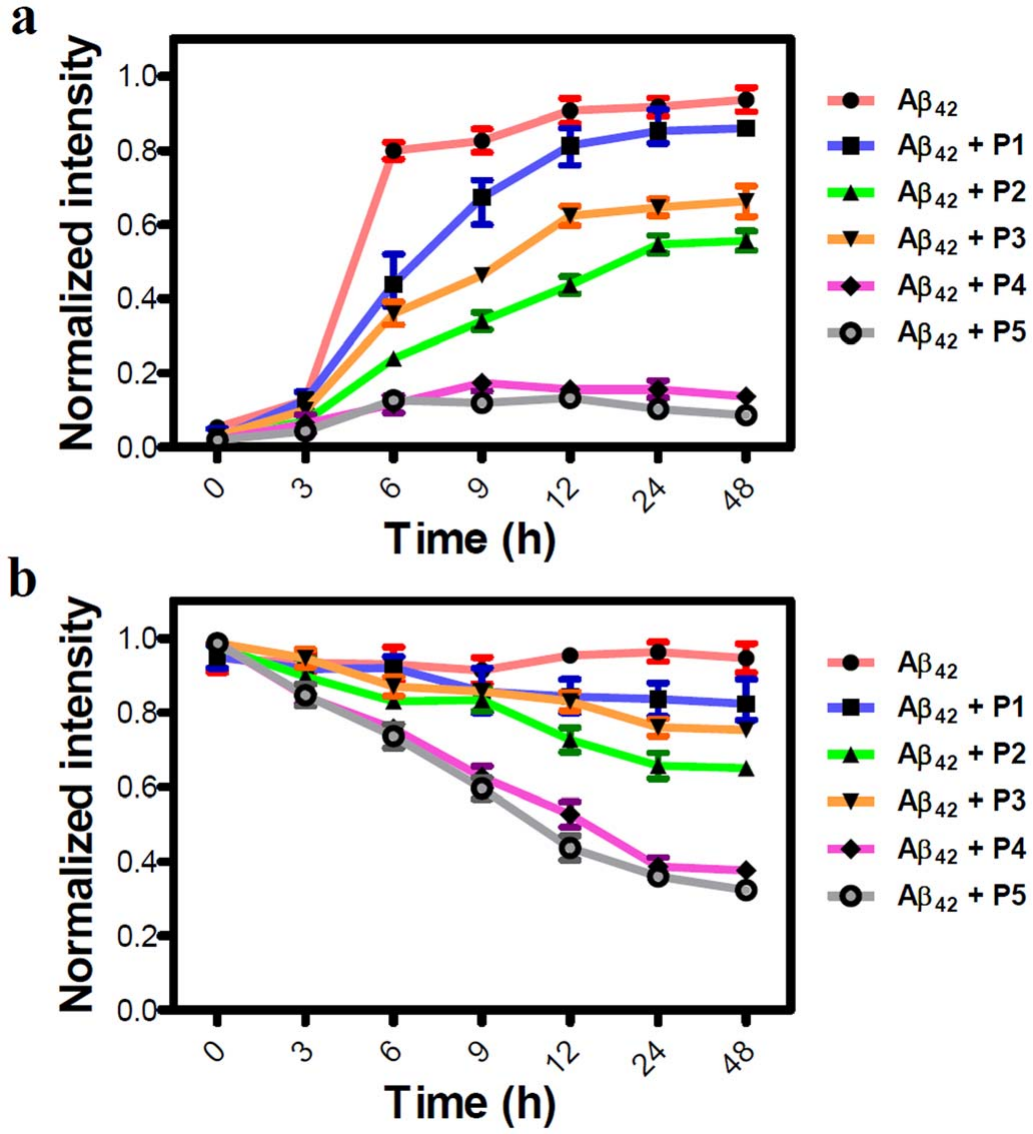
Kinetics of P1-P5 on inhibition and reversal of Aβ42 fibril using ThT assay. A 20 μM of Aβ42 monomers (a) or their aggregates *(*b) were incubated with inhibitors (**P1**, **P2**, **P3**, **P4** and **P5)** at 37°C in 1:2 stoichiometry and their influence on fibrillization or dissolution is quantified by measuring ThT fluorescence intensity, which is represented as normalized fluorescence intensity at 485 nm for a given time point. Each experiment was repeated three times (n = 3). Error bars represent the standard deviation (SD) of the fluorescence measurement.

Next, we performed time-dependent assays to monitor the effect of inhibitors on the growth kinetics of Aβ42 monomers to fibrillar aggregates and dissolution of toxic aggregates. A sigmoid growth curve was obtained for Aβ42 fibrillization, which has been well-reported in the literature^6^. **P1** showed a slight variation in the growth curve, indicating least effect on the Aβ42 aggregation, whereas **P2** and **P3** showed decreased growth phase to < 60%, signifying moderate inhibition efficiency. **P4** and **P5** were most competent among all the candidates with an inhibition efficiency of > 90% as shown in Fig. 3. During the growth phase, Aβ42:**P4**/**P5** complex showed a slight enhancement in fluorescence (9 h), which decreased at further time points indicating that Aβ42 aggregates formed at a faster rate during the growth phase, but at further time points inhibitors managed to dissolve the aggregates and showed a decrease in fluorescence^20^. In time-dependent reversal assays, a similar order of efficiency was observed where **P1**/Aβ42 complex showed a slight decrement in fluorescence and **P2** and **P3** were moderately active in dissolving the Aβ42 aggregates with efficiencies of 35% and 25%, respectively. **P4** and **P5** again exhibited best dissolution efficiencies of 68% and 75% on the Aβ42 aggregates. To further validate the inhibition efficiency of our most efficient inhibitor **P5**, we performed dot blot analysis in a time dependent manner using specific antibody for Aβ42 aggregates^30^. Aβ42 (20 μM) aggregates were incubated with **P5** in 1:2 stoichiometry and their influence on the dissolution is quantified at different time points (6, 12, 24 and 48 h) by measuring chemiluminescence intensity (supplementary Fig. S1). The dot blot analysis data clearly supported our ThT dissolution assay of Aβ42 aggregates with **P5** as shown in Fig. 3b.

**Figure 4:**
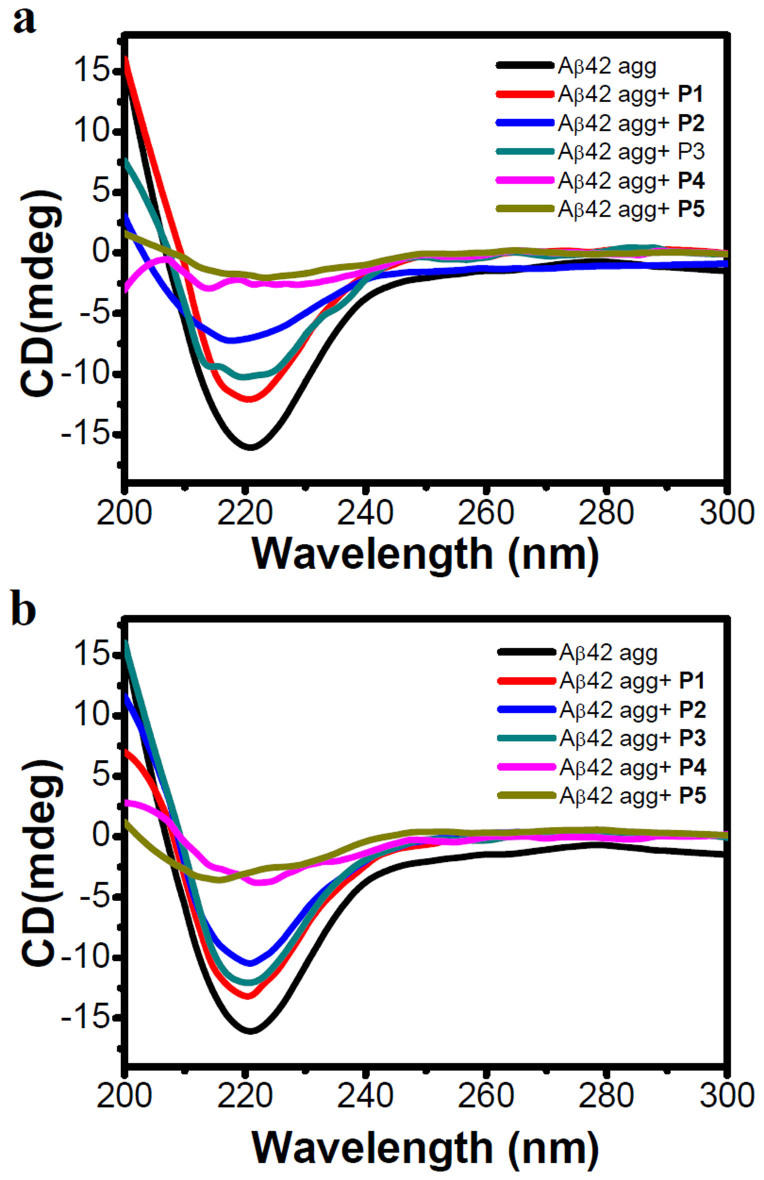
Studying Inhibition and reversal assay of Aβ42 aggregates by CD measurements. The data in (a) and (b) show the effects of **P1**, **P2**, **P3**, **P4** and **P5** (40 μM) on the aggregation of 20 μM Aβ42 (on day 4 for the inhibition assay and day 6 for the reversal assay). Insets in (a) and (b) shows the intensity of negative signal at 218 nm (represents β-sheet content) observed in corresponding experiments. **P4** and **P5** effectively decreased the β-sheet content corresponding to Aβ42 aggregates compared to **P1**-**P3**.

To further validate our results, we performed CD studies. Aβ42 aggregates are predominantly made of β-sheet assembly, which can be assessed by CD measurements. Hence, a decrease in β-sheet content and corresponding characteristic CD signal intensity directly correlate with the inhibition efficiencies of inhibitors. In this assay, we monitored the intensity of negative CD band centered at 218 nm, characteristic of a β-sheet structure and a decrease in its intensity was correlated with a reduction in the toxic Aβ42 aggregates (supplementary Fig. S2). In these CD measurements, samples similar to ThT fluorescence assays were used to allow for direct comparison between the two experiments. In the case of inhibition assay, Aβ42 monomers were incubated with inhibitor candidates for 4 days at 37°C and then CD measurements were performed to evaluate the inhibition efficiency. Aβ42/**P1** mixture showed a slight decrease in β-sheet content (deduced by a decrease in negative band at 218 nm). Aβ42/**P2** showed a slight blue shift and > 50% reduction in CD intensity (a decrease in intensity at 218 nm compared to the Aβ42 control, **P1**) as compared to untreated Aβ42 while **P3** showed only ~30% decrease in β-sheet content. Supporting our results obtained in the ThT assay, **P4** and **P5** emerged as efficient inhibitors as they exhibited > 80% inhibition (corresponding to a decrease in CD band intensity at 218 nm) of the formation of Aβ42 aggregates (Fig. 4). In the reversal assay, Aβ42 aggregates were incubated with the inhibitor candidates for six days at 37°C and then, CD measurements were performed. CD data was in excellent agreement with the dissolution efficiency obtained in reversal assay experiments monitored by the ThT assay (Fig. 2). **P2** and **P3** showed moderate dissolution efficiencies of 40% and 25%, respectively towards the Aβ42 aggregates. **P4** and **P5** showed appreciable dissolution efficiencies of > 75% (Fig. 4). Overall, inhibition and dissolution efficiencies obtained in the ThT fluorescence assay and CD measurements were in good agreement.

To prove that the above results were purely due to changes in Aβ42 and had not been altered by the self-aggregation of inhibitory peptides, we performed a time-dependent assay over a period of 10 days where all the inhibitor candidates (**P1**- **P5**) were incubated at 37°C and their effect on the fluorescence of ThT was monitored. Fluorescence enhancement shown by inhibitor (**P1**- **P5**) alone was almost negligible, which was further confirmed by CD measurement, even on the tenth day of incubation modulator peptides did not adopt any secondary conformations (supplementary Fig. S3)^30^.

### TEM Analysis

**Figure 5:**
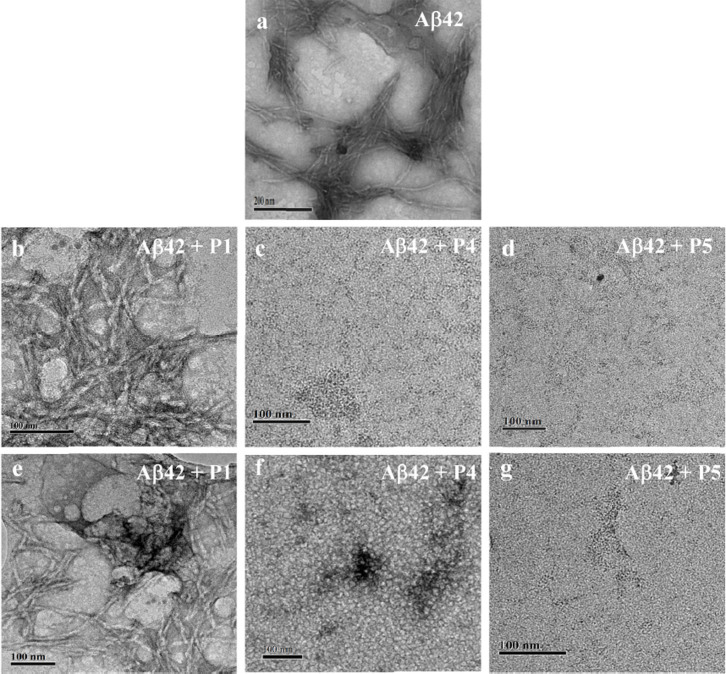
Electron microscopy examination to study the effect of P1, P4 and P5 on Aβ42 fibrillization and dissolution of aggregates. **P1, P4** and **P5** were incubated with 20 μM of Aβ42 monomers or their aggregates at 37°C in 1:2 (Aβ42:inhibitor) stoichiometry and analyzed on day 6 for the inhibition assay and day 12 for the reversal assay experiments. Inhibition assay: Aβ42 a, Aβ42 + **P1** b, Aβ42 + **P4** c, and Aβ42 + **P5** d. Reversal assay: Aβ42 + **P1** e, Aβ42 + **P4** f, and Aβ42 + **P5** g. P1 showed least effect on morphology of Aβ42 fibrils, whereas **P4** and **P5** showed prevention of Aβ42 fibrils formation in inhibition assay and dissolution of Aβ42 aggregates in reversal assay. Scale bar: 100 nm.

To further consolidate our conclusions drawn from the ThT assay and CD measurements, we performed TEM to analyze the effect of **P4** and **P5** on the process of fibrillization and preformed toxic aggregates of Aβ42 (Fig. 5). All the experiments were performed with 20 μM of Aβ42 in PBS buffer (10 mM, pH 7.4). For inhibition experiments, Aβ42 monomers were incubated with **P4** or **P5** for 6 days at 37°C while for reversal experiments preformed Aβ42 aggregates were incubated with **P4** or **P5** for 12 days at 37°C. Aβ42, when incubated in PBS buffer at 37°C for two days, showed the presence of long fibrillar aggregates (Fig. 5a). **P1** was used as a negative control as it did not show any significant changes in the inhibition or dissolution experiments as monitored in the ThT assay and CD measurements. **P1** incubated with Aβ42 showed the presence of fibrillar aggregates in both inhibition and reversal experiments as shown in Figures 5b and 5e, respectively. In contrast, **P4** showed complete absence of fibrils in the inhibition experiment confirming the prevention of fibrillar growth of Aβ42 (Fig. 5c). In the case of reversal experiment, there were no signs of fibrils in **P4** (Fig. 5f). Similarly, **P5** showed the absence of fibrillar aggregates in inhibition (Fig. 5d) and reversal (Fig. 5g) experiments. Further, the observed globular morphology of **P4** treated Aβ42 fibrillar aggregates as shown in Fig. 5f could be misunderstood as toxic oligomeric species. To investigate this, we performed a dot blot analysis where Aβ42 (20 μM) aggregates were incubated with **P4** and **P5** in 1:2 (Aβ42: inhibitor) stoichiometry for 12 days at 37°C and then treated with A11 antibody (which specifically binds to toxic Aβ42 oligomeric species) followed by treatment with secondary antibody and finally chemiluminescence intensity was measured. Positive control, Aβ42 oligomers showed a signal, whereas Aβ42+ **P4** and Aβ42+ **P5** did not show any signal indicating absence of toxic oligomeric species. To further verify the findings, toxicity assay was performed where yeast cells (*Saccharomyces cerevisiae*) were incubated with Aβ42 oligomers (50 μM) and Aβ42 (50 μM) aggregates which were treated with **P4** in 1:2 (Aβ42:**P4**) stoichiometry, at 37°C and their effect on the growth curve was monitored. Aβ42 oligomers showed high toxicity (Fig. S5), whereas Aβ42+**P4** sample showed least effect on the growth curve of yeast cells. Therefore, the dot blot analysis and toxicity assay confirms that globular structures seen in Fig. 5f are not toxic Aβ42 oligomeric species (supplementary Fig. S4 and Fig. S5)^41^. Therefore, TEM data confirmed that **P4** and **P5** were involved in the inhibition and dissolution of toxic aggregates, which is in agreement with the conclusions drawn from other experiments.

### Blood plasma and proteolytic stability for peptiomimitics

**Figure 6:**
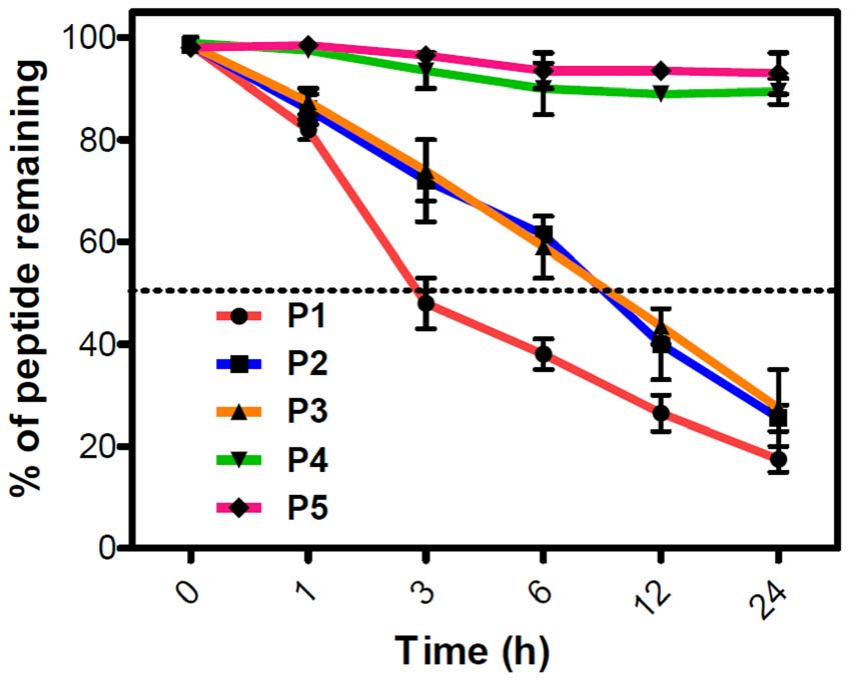
Serum stability of inhibitors. Inhibitors (50 μM) (**P1**-**P5)**were incubated in human blood serum (HBS) and were analyzed at different time points over a duration of 24 h to determine the percentage of intact inhibitor. **P1**, **P2** and **P3** degraded with time, whereas **P4** and **P5** showed high stability towards the serum proteases. Each experiment was repeated three times (n = 3). Error bars represent the standard deviation (SD) of the fluorescence measurement.

The impact of N-terminal modification (**P2** and **P3**) and Sr (N-methylglycine) substitution (**P4** and **P5**) in **P1** were investigated for their proteolytic stability towards blood plasma proteases^37^. The assay involved the incubation of peptides (50 μM) with blood serum at 37°C for a period of 24 h and assessing the amount of intact peptides at different time points (0, 3, 6, 12 and 24 h) using RP-HPLC. **P1** exhibited greatest susceptibility towards the serum proteases with a serum half-life of ~3 h, and 80% of the **P1** was degraded at 24 h (Fig. 6). In contrast to that, **P2** and **P3** with modified N-terminal of **P1** (thymine and barbiturate, respectively) showed a better protease stability towards blood plasma. Both **P2** and **P3** followed almost a similar path of degradation with time, where they showed a half-life of ~10 h. This was 3 times higher compared to **P1** indicating that the N-terminal modification with a non-amino acid moiety had enhanced the blood protease stability by interfering with the degradation ability of proteases. At 24 h, both **P2** and **P3** were degraded to 70%, which was comparable to **P1** suggesting that stability of both **P2** and **P3** decreased with time. Remarkably, **P4** and **P5** were very stable towards blood plasma proteases in comparison to the other peptides (**P1**, **P2** and **P3**). After 24 h, more than 90% of **P4** and **P5** were intact with **P5** exhibiting relatively higher stability than **P4** (Fig. 6). Proteolytic enzymes generally recognize the specific amide bond between the natural amino acids and cleave them. In the case of **P1**, **P2** and **P3** all the amino acids were natural (except N-terminal modifications in **P2** and **P3**, which showed marginally higher stability) and could be easily recognized by proteases; these peptides thus, degraded with time. On the other hand, **P4** and **P5** with an unnatural amino acid (Sr: N-methylglycine) in alternate positions were not recognized by the blood plasma proteases, resulting in their high blood plasma protease stability.

To further validate these results, we performed a stability assay for **P1**-**P5** (50 μM) in the presence of proteolytic enzymes trypsin and pepsin. Enzymes trypsin and pepsin are well-known to cleave the C-terminal of lysine and amide bond involving aromatic amino acids, respectively^39^. Peptides were incubated with both the enzymes at 37°C for 24 h, and the amount of the residual intact peptide was monitored in each case at different time points (0, 3, 6, 12 and 24 h) using RP-HPLC. **P1**, **P2** and **P3** were less stable, of which **P1** degradation was fastest followed by **P2** and **P3**. However, **P4** and **P5** were highly stable under similar conditions and > 90% of the peptidomimetics was intact after 24 h of incubation in the presence of both the enzymes suggesting their poor recognition by the two proteases (supplementary Fig. S6). Overall, the stability assays with blood plasma and proteolytic enzymes led to similar conclusions and confirmed the stability, order **P5**>**P4**≫**P3**≈**P2**>**P1**.

### Designed Peptidomimetics NullifyAβ Toxicity in an Autophagy-Dependent Manner

**Figure 7:**
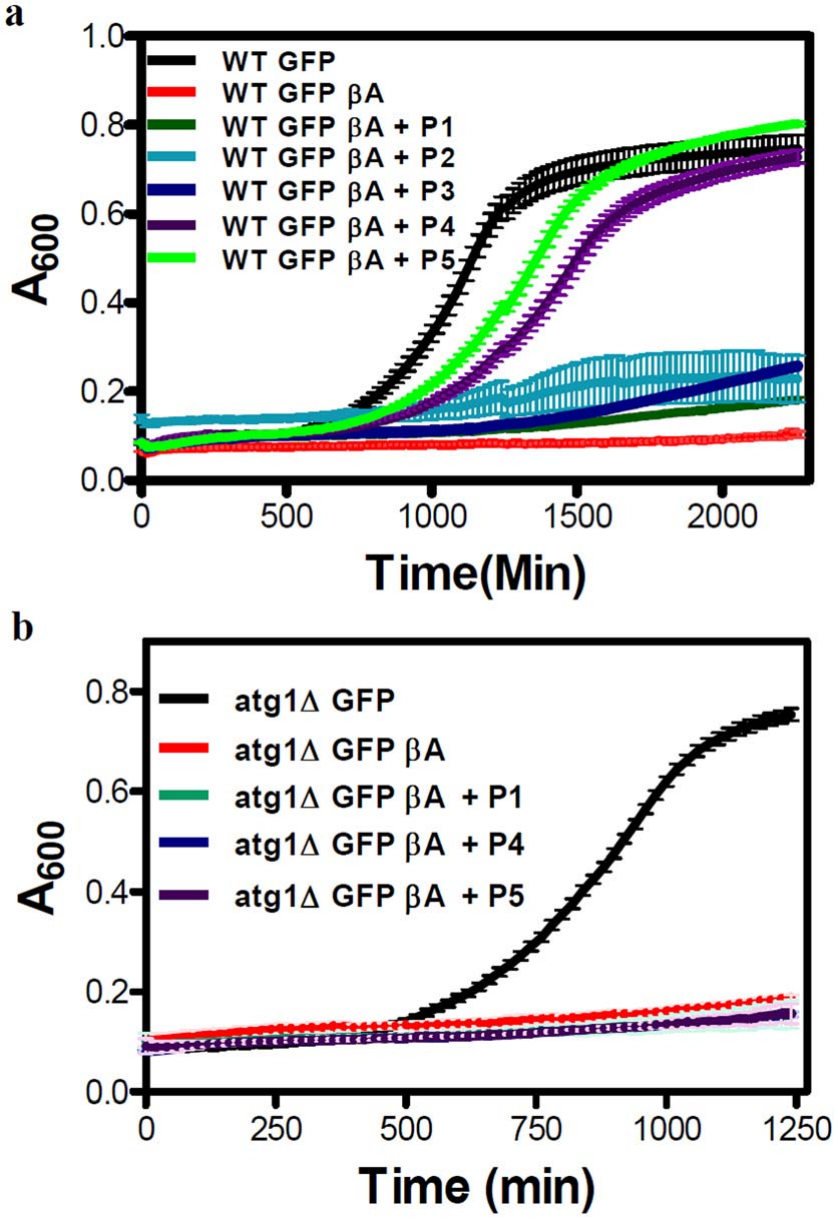
Screening of inhibitors in a yeast model of Aβ toxicity. (a), All inhibitor candidates were screened in WT GFP βA strain at the concentration of 300 μM. (b), Investigation of **P1**, **P4** and **P5** in the *atg1Δ* GFP βA strain by monitoring growth curves at peptide concentrations of 300 μM. Each experiment was repeated three times (n = 3). Error bars represent the standard deviation (SD) of the measurement.

All the peptides and their analogs (**P1-P5**) were screened for their ability to ameliorate the toxicity caused by Aβ42 in a *Saccharomyces cerevisiae* model. N-terminal of Aβ42 was tagged with GFP (WT GFP βA) while the WT GFP strain was used as a control. To study the non-toxic nature of inhibitor candidates, their influence on culture growth curves of WT GFP were analyzed (supplementary Fig. S7). In **P1**-**P5** (300 μM) treated cells, the growth curves were similar to that of the untreated sample. No significant growth lag or drop in absorbance (A_600_) was observed in the presence of peptides. On the other hand, the growth curve of WT GFP βA exhibited a severe lag with the culture not entering the exponential phase due apparently to Aβ toxicity^36^. The apparent growth lag displayed by WT GFP βA strain compared to WT GFP was used for screening the inhibitors (Fig. 7a). Among five inhibitors, growth curves of WT GFP βA strain in the presence of peptides **P1**, **P2** and **P3** appeared similar to that of untreated cells. However, the cells treated with peptides **P4** and **P5** displayed a growth pattern similar to that of WT GFP. Hence, it is inferred that peptides **P4** and **P5**, but not the others, successfully rescued the growth lag in WT GFP βA strain. Upon **P4** and **P5** treatments, various growth parameters like growth rate and doubling time in WT GFP βA strain showed significant rescue comparable to that of WT GFP were the growth rate was increased whereas doubling time was reduced evidently (supplementary Fig. S8).

**Figure 8:**
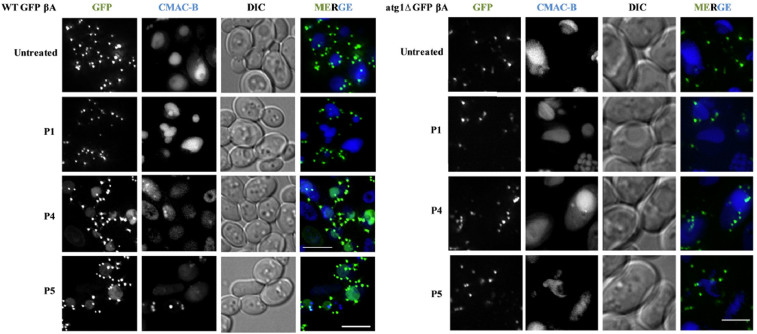
Degradation of GFP Aβ in monitored by fluorescence microscope. (a),WT GFP βA and (b), atg1 βA strains were treated with **P1** (control), **P4** and **P5** and vacuoles stained with CMAC-Blue Scale bars: 7.5 μm (a) and 5 μm (b). Concentration of **P1**/**P4**/**P5**: 300 µM.

Next, we probed if the inhibitors were able to clear the Aβ aggregates (tagged with GFP) *in vivo*. For this, we performed a microscopy assay wherein the GFP βA appear as punctate dots when present as aggregates while its clearance in the vacuole is marked by the presence of free GFP. GFP βA-expressing cells, when either untreated or treated with peptide **P1,** displayed characteristic punctate formation inside the cells and no free GFP was present in the vacuole (Fig. 8a). The free GFP was observed in vacuoles in culture treated with **P4** and **P5,** but was absent in **P1** (control) and untreated cells (Fig. 8a) of WT GFP βA strain. Pertaining to **P4** and **P5**, diffused GFP signal in the vacuole co-localized with the vacuolar lumen staining dye CMAC-Blue, suggesting that incubating cells with these peptides resulted in GFP βA aggregates being degraded in the vacuole to release free GFP. Autophagy has been shown to play a key role in degrading the β-amyloid oligomers or fibrils^42^. Degradation of protein aggregates by selective autophagy mechanism is defined as aggrephagy^43^. In cellular context, the disaggregated fibrils are captured and released into the vacuole for degradation through autophagy. To investigate whether the appearance of free GFP in the vacuoles of cells treated with **P4** and **P5** was due to autophagy, we repeated the growth rescue experiment in cells defective in autophagy (Δatg1 mutant). Although **P4** and **P5** were able to rescue the growth lag in WT GFP βA strain, they failed to do so in *atg1*Δ GFP βA strain (Fig. 7b). In addition, vacuolar free GFP was not seen in *atg1*Δ GFP βA strain treated with **P4** and **P5** (Fig. 8b) similar to untreated and **P1** treated cells. The peptides neither reduced the Aβ toxicity nor degraded GFP βA in the autophagy mutant. This clearly indicated that autophagy was responsible for clearing the Aβ aggregates, by **P4/P5** treatments in WT GFP βA cells.

## Conclusion

In conclusion, we rationally designed recognition unit based peptidomimetic inhibitors, which target hydrogen bonding and other noncovalent interactions necessary for Aβ aggregation to form toxic aggregates in Alzheimer's disease progression. ThT fluorescence assay and CD data confirmed that peptides with N-terminal thymine-modification (**P2**) and peptidomimetics containing N-terminal thymine and sarcosine (N-methylglycine) in alternate positions of KLVFFA (**P4** and **P5**) exhibited both inhibition and dissolution ability towards Aβ42 aggregates with the latter two being considerably more efficient. TEM analysis demonstrated that **P4**/**P5** treated Aβ42 monomers or its aggregates showed no signs of fibrillar aggregates compared aggregates found in control study, which further strengthen our hypothesis that **P4** and **P5** are involved in inhibition and dissolution of Aβ42 aggregates. Furthermore, peptidomimetics **P4** and **P5** showed high stability towards blood serum and proteolytic enzymes like trypsin and pepsin compared to **P1**-**P3**. Therapeutic contenders **P4** and **P5** were tested in a *Saccharomyces cerevisiae* model of Aβ42, where they could rescue the yeast cells from Aβ42 toxicity by clearing them through the autophagy pathway. Although down-regulation of autophagy is implicated in Alzheimer's disease, for the first time we validated the role of active autophagy in clearance of toxic Aβ aggregates using peptidomimetics. These results on rationally designing peptidomimetic inhibitors for tackling Aβ42 toxicity in Alzheimer's disease will strongly impact the identification of novel drug candidates for this hitherto incurable disease.

## Methods

### Synthesis of Peptide and its Mimetics, Purification, and Analysis

The control peptide **P1** (KLVFF), N-terminal modified peptides **P2** (Thymine-Lys-Leu-Val-Phe-Phe) and **P3** (Barbiturate-Lys-Leu-Val-Phe-Phe), and the N-methyl glycine (sarcosine: Sr) substituted peptidomimetics **P4** (Thymine-Sr-Leu-Sr-Phe-Sr-Ala) and **P5** (Thymine-Lys-Sr-Val-Sr-Phe-Sr) were synthesized following standard 9-fluorenylmethoxycarbonyl (Fmoc) chemistry on an automated peptide synthesizer Syro II from MultiSynTech. Rink amide resin (Novabiochem) was used as a solid support in the synthesis with an amide at the C-terminal. Fmoc-protected sarcosine (Sr) was prepared using standard protection procedure and directly used for the synthesis of **P4** and **P5** using the peptide synthesizer. Amino acids were coupled using HBTU as the activating reagent, DIPEA as the base and DMF as solvent; for deprotection of Fmoc 40% piperidine in DMF was used. **P1**, **P2** and **P3** were synthesized with a coupling time of 1 h per amino acid, whereas for **P4** and **P5** coupling time was increased to 2 h to obtain higher coupling yields. All the peptides and peptidomimetics were purified using a reverse-phase (RP) preparative HPLC on C18 column at 40°C. Product purity was greater than 99% as ascertained by analytical HPLC. The molecular masses of the peptides and their mimetics were verified with HRMS (Q-TOF) analysis.

### Preparation of Aβ42 fibrillar aggregates and oligomers

Aβ42 peptide (0.25 mg) (Calbiochem, Merck) was dissolved in hexafluoro-2-propanol (HFIP, 0.2 mL) and incubated at room temperature for 1 h. HFIP was then removed by the flow of nitrogen and further dried under vacuum. HFIP-treated Aβ42 was then dissolved in 10 mM PBS buffer to a concentration of 200 μM at pH 7.4. The solution was incubated at 37°C for 48 h with constant shaking for fibril formation. The formation of Aβ42 fibrillar aggregates was confirmed by ThT fluorescence, CD measurements and electron microscopy. For oligomers, HFIP-treated Aβ42 dissolved in 10 mM PBS buffer and incubated at 4°C for 24 h^44^.

### Fluorescence Spectroscopy

Fluorescence spectral measurements were carried out using Perkin Elmer Model LS 55 fluorescence spectrophotometer. Maximum fluorescence of ThT was observed with the excitation and emission wavelengths set to 450 and 483 nm, respectively. A ThT concentration of 5–10 µM was used for amyloid fibrillization and dissolution assay based on the Aβ42 fibrillar concentration.

### Circular Dichroism

The circular dichroic (CD) spectra were recorded using a Jasco J-815 spectrometer under nitrogen atmosphere. Peptides were dissolved in 10 mM PBS buffer at pH 7.4 at concentrations of 20 µM. A 10 mm path length was used for the measurements. Four to five scans were acquired from 200 to 300 nm.

### Fibrillogenesis and Fibril Dissolution Assays

Prior to the experiment, **P1**, **P2, P3, P4** and **P5** were dissolved in HFIP and were evaporated under a stream of dry nitrogen. The dried samples were re-suspended in 10 mM PBS buffer at pH 7.4. An aliquot of Aβ42 peptide was then added to the solution with or without one of the inhibitor **P1**, **P2, P3, P4** and **P5**. The mixtures were vortexed for approximately 30 s and then incubated at 37°C for 2–3 days without shaking. The final concentration of Aβ42 in the mixture was 20 μΜ. For a dissolution experiment, Aβ42 was incubated alone for 2 days to allow fibrils to form. An aliquot of the formed fibrils in 10 mM PBS buffer was then added to the inhibitor peptidomimetics. The amount of fibrils remaining intact was assayed using that fluorescence, CD measurements and transmission electron microscopy as described below.

### Transmission Electron Microscopy (TEM)

An aliquot of appropriately formed samples of Aβ42 aggregates, Aβ42-inhibitor and Aβ42 aggregate-inhibitor (5 μL) were adsorbed onto 200-mesh carbon and formavar-coated grids for 2 min and washed for 1 min with distilled water. The samples were negatively stained with 2% uranyl acetate for 5 min and washed for 1 min with distilled water^44^. The samples were air-dried overnight and viewed with a JEOL, JEM 3010 instrument operating at 300 kV.

### Dot Blot Analysis

PVDF membranes (Sigma aldrich) were activated by incubating them in methanol solution for 5 min followed by washing with 10 mM PBS buffer (3X). Samples were spotted on the membranes (in triplicate) and non-specific sites were blocked by soaking in 5% BSA in PBS buffer, and skim milk (0.5–1 hour, RT). Then membranes were incubated with either primary antibody A11 (1:3000) for oligomer or Anti-beta-amyloid 1-42 antibody (Merck millipore) for Aβ42 fibrillar aggregates at 4°C for overnight and then washed with PBS buffer (3 × 5 min). These membranes were further incubated with anti-mouse secondary antibody (1:10000) conjugated with horseradish peroxidase (HRP) for 30 min at RT. These membranes were washed with PBS buffer (3 × 5 min), incubated with enhanced chemiluminescence (ECL) reagent for 1 min and recorded the chemiluminescence in SYNGENE G-box. The signals from the unknown samples were compared to that of standard and concentration was estimated^30^.

### Serum Stability Assay

Human blood serum (HBS) was used to determine serum stability of the inhibitors. The HBS was centrifuged to remove the lipid component, and the supernatant was incubated at 37°C. 50 Μm of each inhibitor (**P1**, **P2, P3, P4** and **P5**) was incubated in HBS and then 40 μL triplicate aliquots were removed at 0, 1, 3, 6, 10, 16, and 24 h. Each serum sample was quenched with 40 μL of 6 M urea and incubated at 4°C for 10 min. Then, each serum sample was quenched with 40 μL of trichloroacetic acid (20%) and incubated for another 10 min at 4°C to precipitate the serum proteins. The samples were centrifuged for 10 min, and 200 μL of the supernatant was analyzed on RP-HPLC using a linear gradient of solvent B (0.3 mL/min flow rate). The control samples containing equivalent amounts of inhibitors in PBS buffer were subjected to the same treatment procedure. The percentage recovery of inhibitors was detected by integration at 254 nm^45^.

### Protease Stability Assay

Preliminary stability assays were performed using the enzymes pepsin and trypsin. For all assays, peptides were incubated with the enzyme at 37°C for 24 h. All digestion assay data were analyzed by RP-HPLC. Trypsin and pepsin stocks were prepared in 100 mM ammonium bicarbonate (NH_4_HCO_3_) buffer (pH 8) and 100 mM formic acid buffer (pH 2), respectively. All the peptides/peptidomimetics (**P1**, **P2**, **P3**, **P4** and **P5**) were incubated with trypsin and pepsin enzymes in 100 mM NH_4_HCO_3_ buffer (pH 8) and formic acid buffer (pH 2), respectively, at 37°C. The trypsin reactions were halted with 0.05% formic acid, and the pepsin reactions were halted with 100 mM NH_4_HCO_3_. Then the samples were analyzed by RP-HPLC using a linear gradient of solvent B (0.3 mL/min flow rate); similar data points were collected at various time points between 0 and 24 h (1 h, 3 h, 6 h, 12 h and 24 h) during incubation and analyzed in triplicates. The percentages of recovered peptide/peptidomimetics were detected by integration at 254 nm^46^.

### Yeast Media, Plasmids and Media Used

Wild type (BY4741; Mat α; his3Δ1; leu2Δ0; met15Δ0; ura3Δ0) and autophagy mutant (*atg1*Δ;BY4741; Mat α; his3Δ1; leu2Δ0; met15Δ0; ura3Δ0; atg1: KanMX4) strains of yeast were employed. Plasmids pRS 416 GFP, pRS 416 βA were gifted by Ian Macreadie (CSIRO, Australia)^47^,^48^. The plasmid pRS 306 Gal βA was generated by sticky end ligation of vector pRS 306 Gal (Spe I/Xho I) and insert GFP- βA (Spe I/Xho I) obtained from the plasmid under pRS 416 vector backbone. These plasmids were used to generate sSNS1, sSNS50 and sSNS51 strains expressing GFP only, GFP-tagged β-amyloid (Aβ) protein in wild type and autophagy mutant, respectively. These strains expressed genomically integrated GFP tagged Aβ or GFP only under an inducible galactose promoter. SD-Ura (Synthetic Dextrose without uracil) media and galactose (2%) were used for protein induction.

### Yeast Culturing and Growth Assay

Strains were inoculated into SD-Ura growth medium and incubated overnight (250 rpm, 30°C). Secondary culture was inoculated (absorbance = A_600 _around 0.2 OD) from the primary inoculum and incubated as above till A_600_ reached 0.8 OD. High throughput growth curve analysis (using Varioskan Flash, Thermo Scientific) in the presence and absence of peptides (300 μM) was performed by automatically recording A_600_ every 20 min in a 384-well plate.

### β-Amyloid Degradation Assay

The cells were inoculated in SD-Ura medium under appropriate conditions (250 rpm, 30°C). Secondary culture was inoculated from this primary inoculum and incubated till A_600_ reached 0.8. Then, cells were washed twice with sterile water. Subsequently, the Aβ proteins were induced in yeast cells by incubating them in 2% galactose. During the induction, these cells were incubated in the presence or absence of peptidomimetics (300 μM). After 12 h, 7-amino-4-chloromethylcoumarin-Blue (CMAC-Blue) was added and incubated further for 2 h after which cultures were imaged on a fluorescent widefield microscope (DV Elite, Deltavision).

### Vacuole Staining by CMAC-Blue

CMAC Blue dye (Life technologies) was used to stain the yeast vacuole. Excitation and emission peaks for CMAC-Blue were 350 nm and 450 nm, respectively. Dye was added to the culture at a final working concentration of 100 nM, incubated for 30 min (250 rpm, 30 µC) and then imaged.

### Fluorescence Microscopy

Cells were washed and mounted on agarose pad (2%) and imaged using Delta Vision Elite widefield microscope with FITC and DAPI filters. The collected images were processed using Axiovision or DV SoftWoRX software. The excitation CWL/BP and emission CWL/BP for filters used were 490/20 and 529/38(FITC), 390/18 and 435/48 (DAPI).

## Author Contributions

K.R. and T.G. designed the project. K.R. undertook the synthesis, photophysical studies and *in vitro* studies of the modulators, S.N.S. and R.M. designed *in vivo* studies. All authors contributed to writing the manuscript.

## Supplementary Material

Supplementary Information Supplementary Information
