# Supplementary material for: Rationally Designed Peptidomimetic Modulators of Aβ Toxicity in Alzheimer's Disease
Source: Sci Rep. 2015 Jan 30;5:8139. doi: 10.1038/srep08139 (PMC4311240; doi:10.1038/srep08139)
Supplement: Supplementary Information [file srep08139-s1.pdf]

Supplementary Information

Rationally Designed Peptidomimetic Modulators of Aβ Toxicity in Alzheimer's Disease

K. Rajasekhar,[1] S. N. Suresh,[2] Ravi Manjithaya*[2] and T. Govindaraju*[1]

[1]Bioorganic Chemistry Laboratory, New Chemistry Unit, Jawaharlal Nehru Centre for Advanced Scientific Research, Jakkur P.O., Bengaluru 560064, Karnataka, India

[2]Molecular Biology and Genetics Unit, Jawaharlal Nehru Centre for Advanced Scientific Research, Jakkur P.O., Bengaluru 560064, Karnataka, India

*Email: ravim@jncasr.ac.in (R.M) and tgraju@jncasr.ac.in (T.G)

a
b

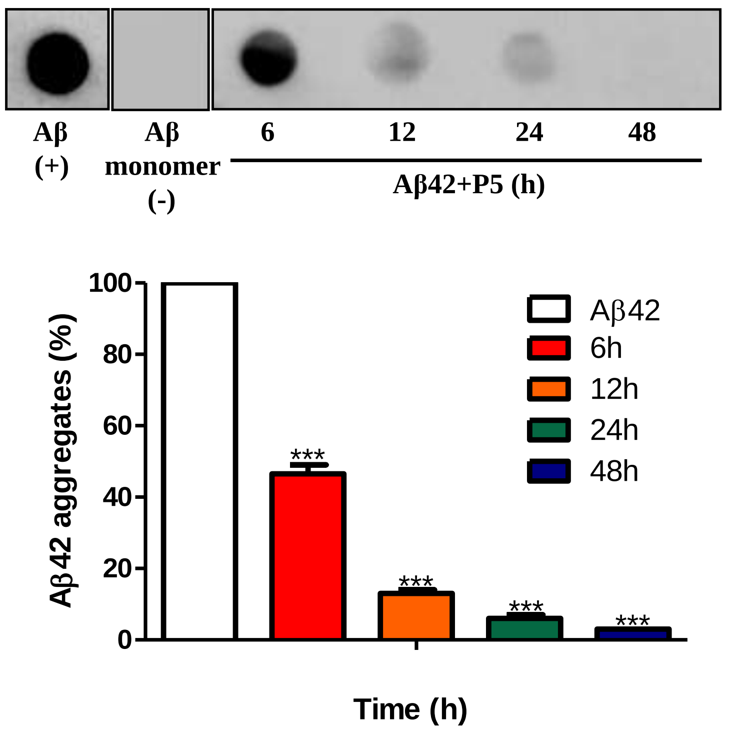

Figure S1. Dot blot analysis to show the dissolution of Aβ42 aggregates using **P5**. (a) Aβ42 (20 μM) aggregates were incubated with **P5** at 37 °C in 1:2 stoichiometry and its influence on dissolution of aggregates is studied at different time points (6, 12, 24 and 48 h) by blotting sample on PVDF membrane and treating it with primary Anti-beta-amyloid 1-42 antibody (1:3000) specific for fibrillar aggregates, followed by anti-mouse secondary antibody (1:10000, conjugated with horseradish peroxidase (HRP)) treatment. The membrane was finally treated with enhanced chemiluminescence (ECL) reagent to measure chemiluminescence and quantify the dissolution of Aβ42 aggregates. (b) Plot of percentage of Aβ42 fibrillar aggregates present at different time point, when compared to control, exact amount of aggregates can be quantified at different time points (For example at 6 h, ~9 μM concentration of Aβ42 aggregates present). Triplicate values for the control were plotted and a difference of 3 standard deviation (SD) units between the test and control was considered as significant. $P<0.05$, ***.

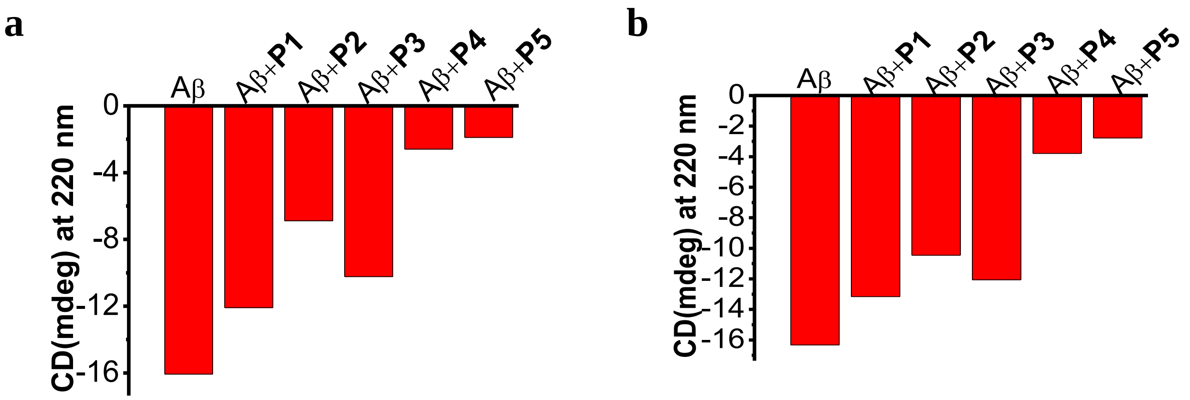

Figure S2. **Inhibition and reversal assay of Aβ42 aggregates by CD measurements**. **a** and **b** show the effects of 1:2 stoichiometry of peptides **P1**, **P2**, **P3**, **P4** and **P5** on the aggregation of 20 μM Aβ42 (on day 3 for the inhibition assay (a) and day 6 for the reversal assay (b)). Were (a) and (b) shows the intensity of negative signal at 218 nm (represents β-sheet content) observed in corresponding experiments. **P4** and **P5** effectively decreased the β-sheet content corresponding to Aβ42 aggregates compared to other three peptides (**P1-P3**).

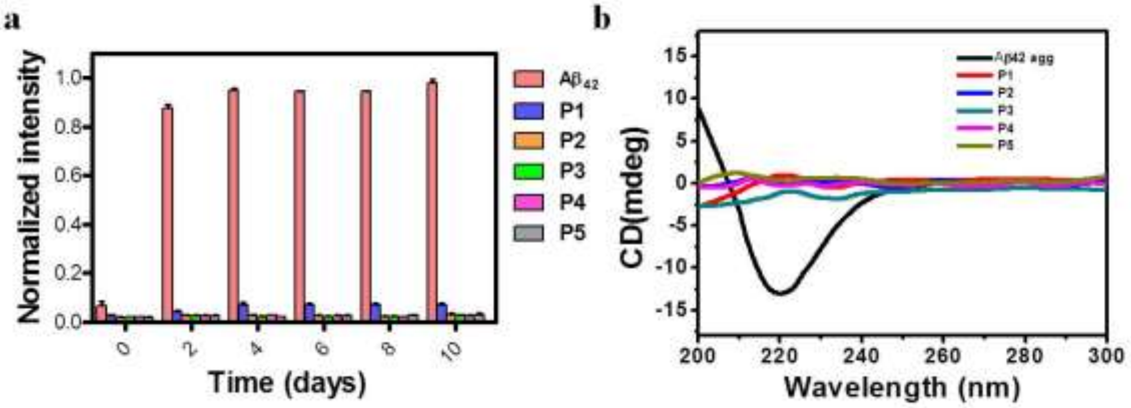

Figure S3. **Thioflavin assay and CD measurements for inhibitor candidates (P1, P2, P3, P4 and P5) incubated in isolation at 37 °C**. **a**, ThT fluorescence was monitored at 485 nm (normalized) for all the peptides (20 μM) in comparison to Aβ42 fibrils (20 μM) from 0 to 10 days. **b**, CD measurement was performed for the same sample used on 10th day of ThT assay, which showed an absence of any distinctive signal corresponding to secondary structure. These data confirmed that **P1**, **P2**, **P3**, **P4** and **P5** are not capable of forming secondary structures by themselves and do not interference with our measurements in ThT and CD measurements.

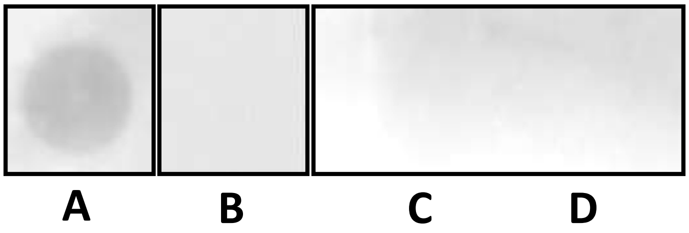

Figure S4. Dot blot analysis of Aβ42 (20 μM) aggregates incubated with **P4** (**C**) and **P5** (**D**) in 1:2 (Aβ42: inhibitor) stoichiometry and incubated at 37 °C. Aβ42 toxic oligomeric species act as positive control (**A**) and Aβ42 (20 μM) monomers as negative control (**B**). A11 (1:3000) is used as primary antibody and anti-mouse antibody (1:10000) conjugated with HRP as secondary antibody and further treated with ECL reagent for 1 min to record chemiluminescence.

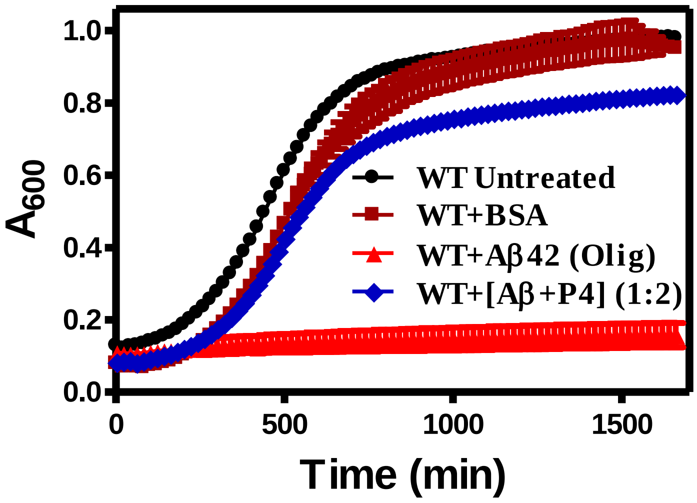

Figure S5. Growth curves of WT yeast cells (*Saccharomyces cerevisiae* BY4741) in presence of Aβ42 oligomers (50 μM) and Aβ42 (50 μM) aggregates treated with **P4** in 1:2 (Aβ42:**P4**) stoichiometry, at 37 °C. Readily formed Aβ42 oligomers incubated with wild type yeast cells showed prominent toxicity, whereas Aβ42+**P4** sample showed least effect on growth curve. This clearly shows that globular structures observed in the TEM for Aβ42+**P4** sample are not toxic oligomeric species.

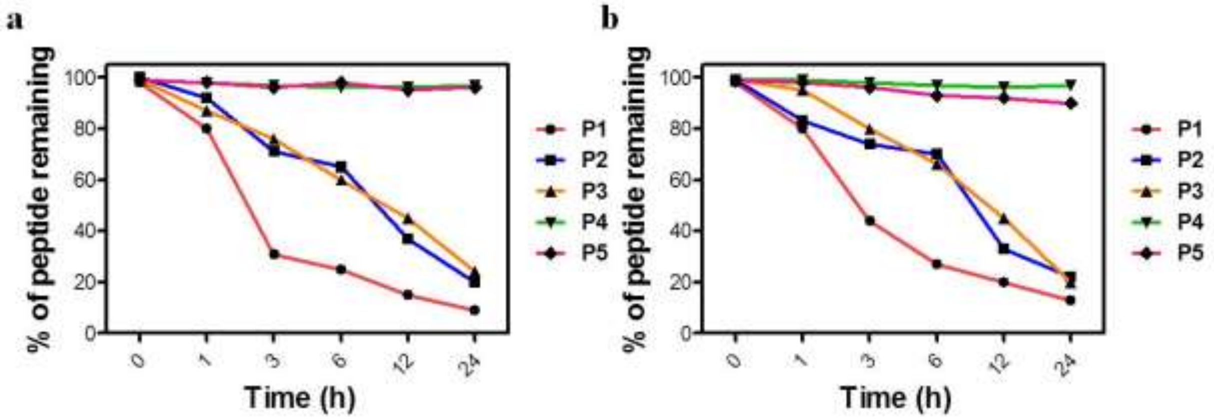

Figure S6. Stability towards protease enzymes pepsin and trypsin. **P1**, **P2**, **P3**, **P4** and **P5** (50 mM) were incubated in with enzymes pepsin **a** and trypsin **b** in 100 mM formic acid buffer (pH 2) and 100 mM NH_4HCO_3 buffer (pH 8) respectively. Percentage of intact peptide in each case was monitored at different time points for a duration of 24 h. **P1**, **P2** and **P3** degraded with time, whereas **P4** and **P5** showed high stability towards protease enzymes.

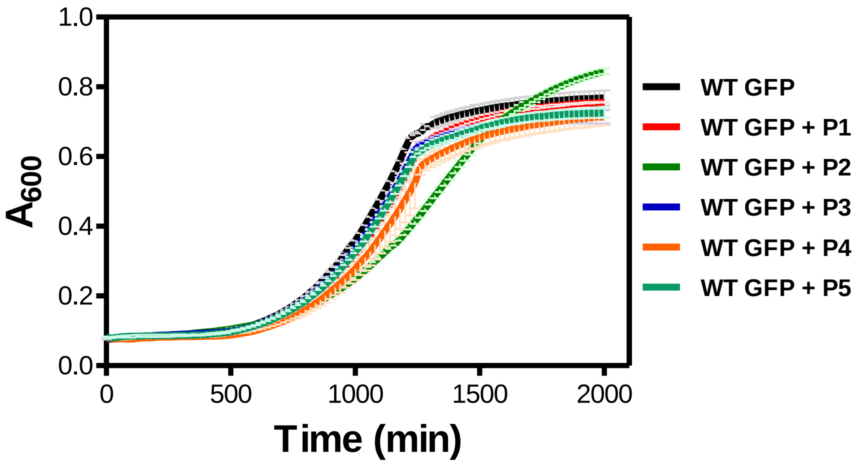

Figure S7. Analysis of peptide toxicities in yeast. Growth curves of WT GFP strain in presence or absence of all peptides were plotted. Concentration of peptides used was 300 µM. Each experiment was repeated three times (n = 3). Error bars represent the standard deviation (SD) of the fluorescence measurement.

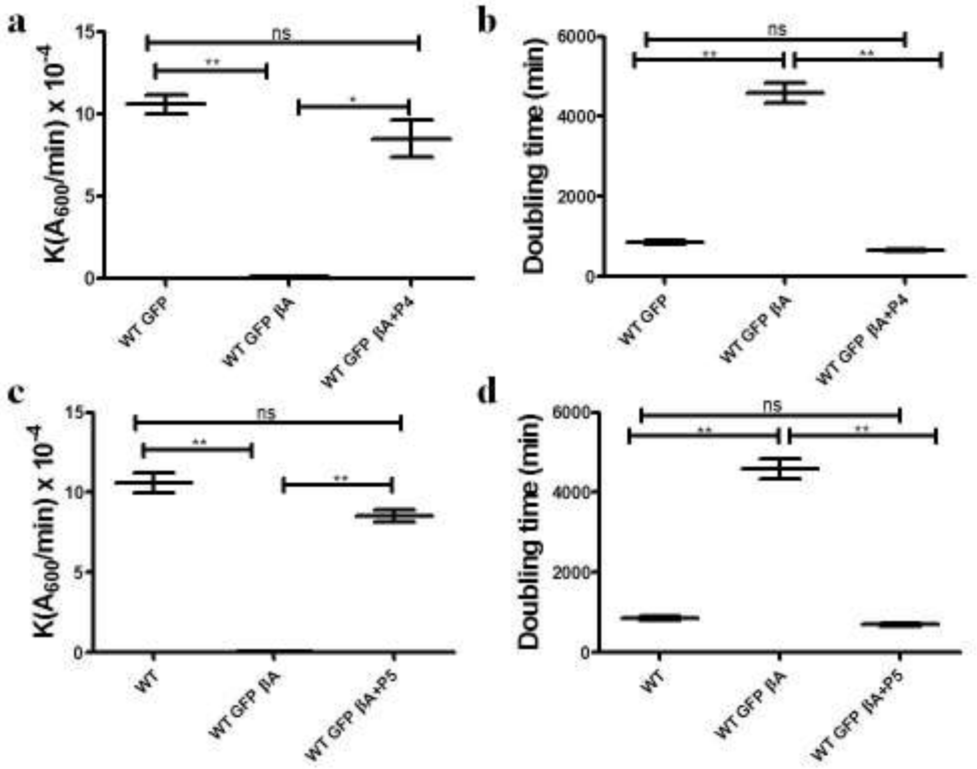

Figure S8. Growth parameters like growth rate and doubling time of WT GFP βA strain in presence and absence of **P4** (**a** & **b**) and **P5** (**c** & **d**) were examined.

HPLC and HRMS traces for P1, P2, P3, P4 and P5

Name	Sequence	Actual mass	Obtained mass $[M+H]^+$
**P1**	Lys-Leu-Val-Phe-Phe	652.4186	652.4170
**P2**	**Thymine**- Lys-Leu-Val-Phe-Phe	818.4565	818.4581
**P3**	**Barbiturate**- Lys-Leu-Val-Phe-Phe	820.4357	820.4373
**P4**	**Thymine**-**Sr**-Leu-**Sr**-Phe-**Sr**-Ala	728.3731	728.3716
**P5**	**Thymine**-Lys-**Sr**-Val-**Sr**-Phe-**Sr**	771.4153	771.4146

Sr = Sarcosine (N-methylglycine).

P1

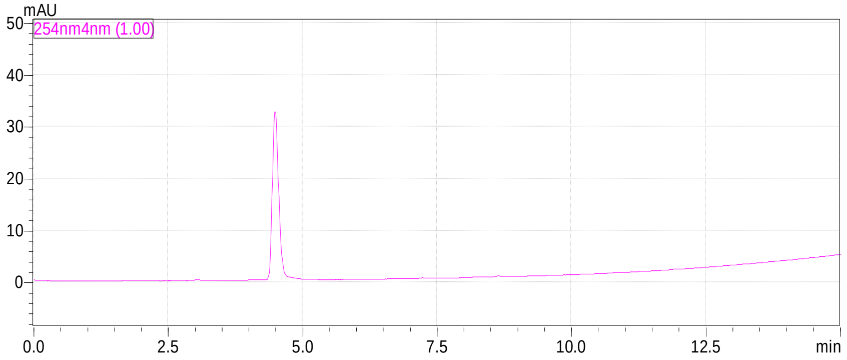

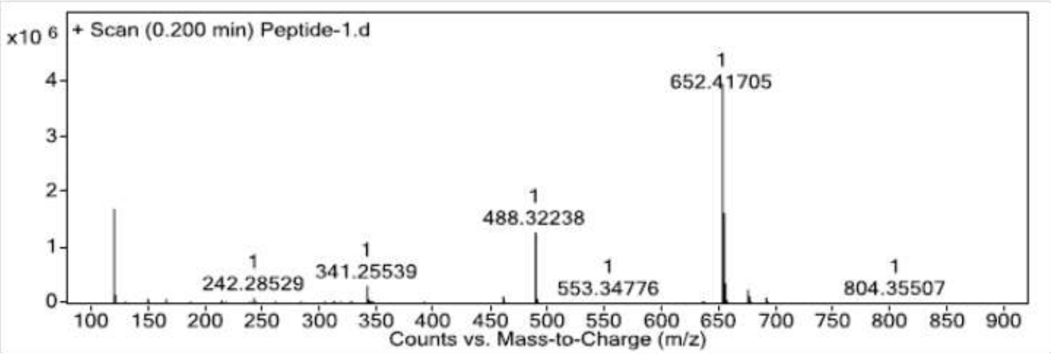

P2

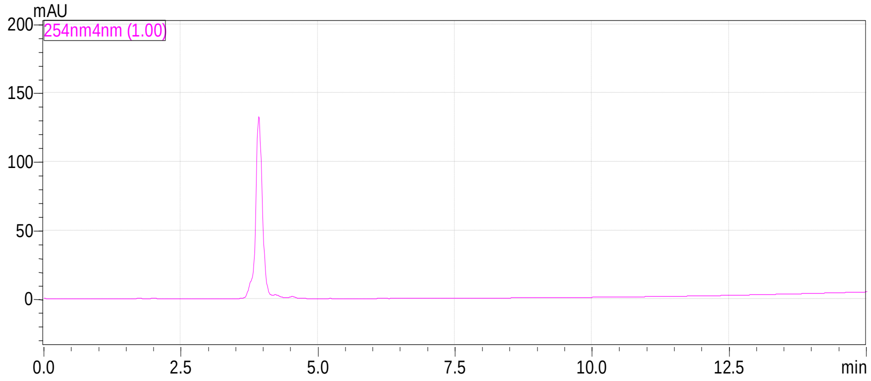
mAU
254nm4nm (1.00)
200
150
100
50
0
0.0
2.5
5.0
7.5
10.0
12.5
min

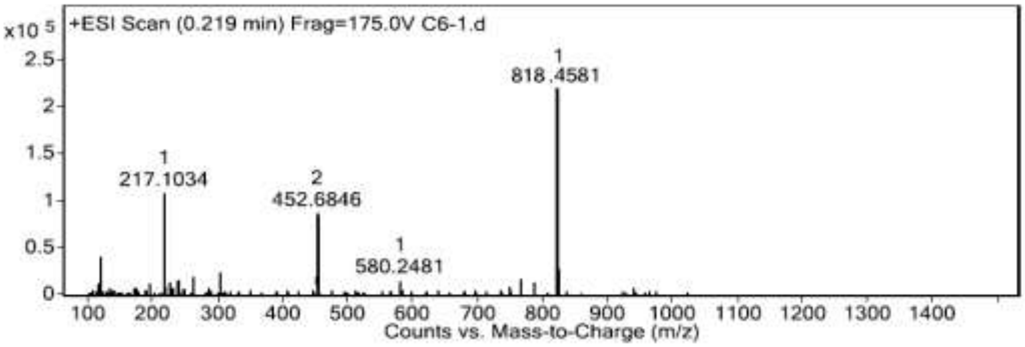
x10 5
+ESI Scan (0.219 min) Frag=175.0V C6-1.d
1
818.4581
1
217.1034
2
452.6846
1
580.2481
2.5
2
1.5
1
0.5
0
100 200 300 400 500 600 700 800 900 1000 1100 1200 1300 1400
Counts vs. Mass-to-Charge (m/z)

P3

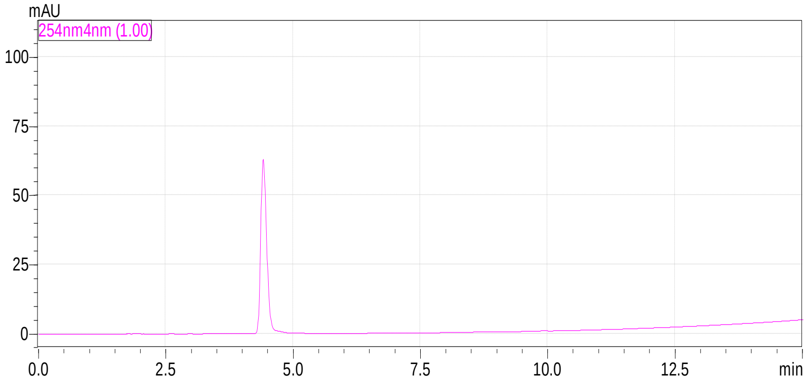
mAU
254nm4nm (1.00)
100
75
50
25
0
0.0
2.5
5.0
7.5
10.0
12.5
min

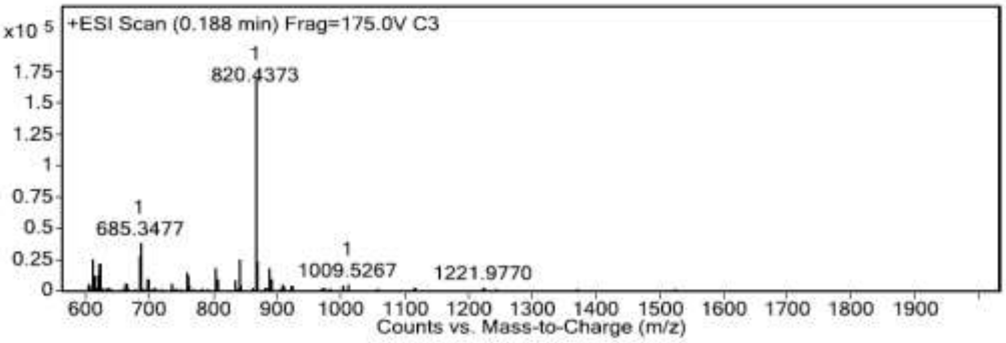
x10 5
+ESI Scan (0.188 min) Frag=175.0V C3
1
820.4373
1
685.3477
1
1009.5267
1221.9770
1.75
1.5
1.25
1
0.75
0.5
0.25
0
600 700 800 900 1000 1100 1200 1300 1400 1500 1600 1700 1800 1900
Counts vs. Mass-to-Charge (m/z)

P4

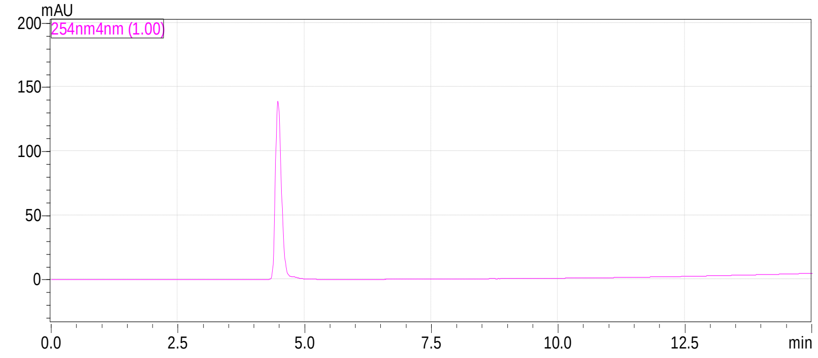
mAU
254nm4nm (1.00)
200
150
100
50
0
0.0
2.5
5.0
7.5
10.0
12.5
min

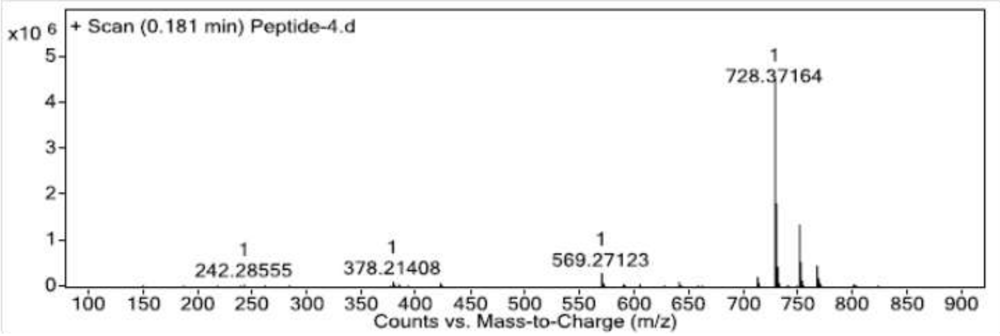
x10 6
+ Scan (0.181 min) Peptide-4.d
1
728.37164
1
242.28555
1
378.21408
1
569.27123
Counts vs. Mass-to-Charge (m/z)

P5

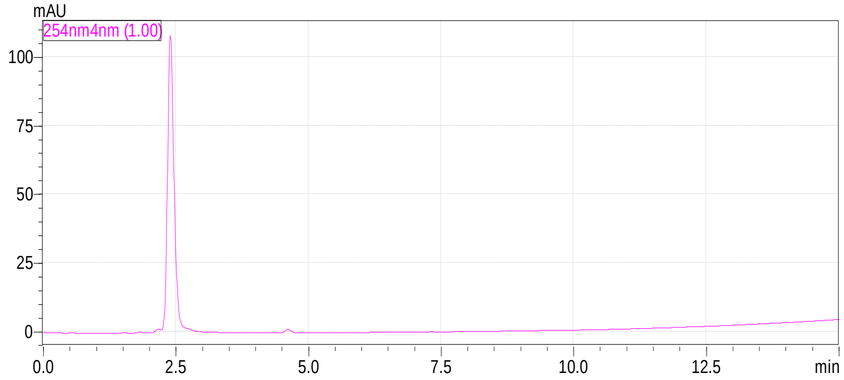
mAU
254nm4nm (1.00)
100
75
50
25
0
0.0
2.5
5.0
7.5
10.0
12.5
min

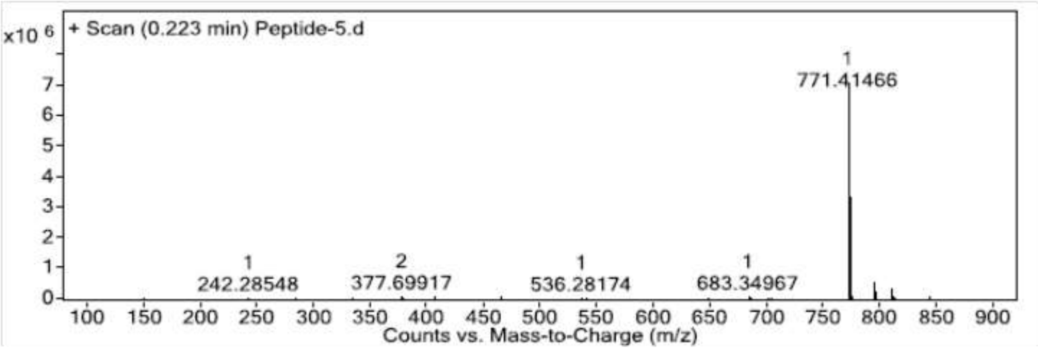
x10 6
+ Scan (0.223 min) Peptide-5.d
1
771.41466
1
242.28548
2
377.69917
1
536.28174
1
683.34967
Counts vs. Mass-to-Charge (m/z)
